# Methodological Quality of Systematic Reviews in Subfertility: A Comparison of Two Different Approaches

**DOI:** 10.1371/journal.pone.0050403

**Published:** 2012-12-28

**Authors:** Ivor Popovich, Bethany Windsor, Vanessa Jordan, Marian Showell, Bev Shea, Cynthia M. Farquhar

**Affiliations:** 1 Faculty of Medicine and Health Sciences, University of Auckland, Auckland, New Zealand; 2 Department of Obstetrics and Gynaecology, University of Auckland, Auckland, New Zealand; 3 Cochrane Menstrual Disorders and Subfertility Group, Department of Obstetrics and Gynaecology, University of Auckland, Auckland, New Zealand; 4 Community Information Epidemiological Communities, University of Ottawa, Ottawa, Canada; Instituto de Efectividad Clínica y Sanitaria – IECS, Argentina

## Abstract

**Background:**

Systematic reviews are used widely to guide health care decisions. Several tools have been created to assess systematic review quality. The measurement tool for assessing the methodological quality of systematic reviews known as the AMSTAR tool applies a yes/no score to eleven relevant domains of review methodology. This tool has been reworked so that each domain is scored based on a four point scale, producing R-AMSTAR.

**Methods and Findings:**

We aimed to compare the AMSTAR and R-AMSTAR tools in assessing systematic reviews in the field of assisted reproduction for subfertility. All published systematic reviews on assisted reproductive technology, with the latest search for studies taking place from 2007–2011, were considered. Reviews that contained no included studies or considered diagnostic outcomes were excluded. Thirty each of Cochrane and non-Cochrane reviews were randomly selected from a search of relevant databases. Both tools were then applied to all sixty reviews. The results were converted to percentage scores and all reviews graded and ranked based on this. AMSTAR produced a much wider variation in percentage scores and achieved higher inter-rater reliability than R-AMSTAR according to kappa statistics. The average rating for Cochrane reviews was consistent between the two tools (88.3% for R-AMSTAR versus 83.6% for AMSTAR) but inconsistent for non-Cochrane reviews (63.9% R-AMSTAR vs. 38.5% AMSTAR). In comparing the rankings generated between the two tools Cochrane reviews changed an average of 4.2 places, compared to 2.9 for non-Cochrane.

**Conclusion:**

R-AMSTAR provided greater guidance in the assessment of domains and produced quantitative results. However, there were many problems with the construction of its criteria and AMSTAR was much easier to apply consistently. We recommend that AMSTAR incorporates the findings of this study and produces additional guidance for its application in order to improve its reliability and usefulness.

## Background

Systematic reviews (SRs) are increasingly used to guide health care decisions. Systematic reviews not only collate findings on a given topic, but also consider the quality of the studies and thus can be considered to be highly reliable. They aim to collect ‘all empirical evidence that fits pre-specified eligibility criteria in order to answer a specific research question’ [1]. Many reviews also incorporate meta-analyses, the statistical combination of findings from individual studies to provide a more precise estimate of the effect of an intervention. The strength of systematic reviews is attributed to their explicit and systematic methodology, consisting of clearly stated objectives, pre-defined eligibility criteria, systematic searching to capture all relevant literature, and assessment of validity and synthesis of findings of included studies.

Despite these objectives, many systematic reviews have been found to be inadequate. A comprehensive report of 300 systematic reviews published in 2007 suggested that the quality of reporting is inconsistent and that readers should not accept SRs uncritically [2]. Large differences were noted between Cochrane (CR) and non Cochrane (NCR) reviews, for example, almost half of the NCRs reported that the significance of the primary outcome was favourable compared to only 14% of the Cochrane reviews. Overestimating the benefits of treatments based on poorly executed and reported SRs could lead to biased recommendations and poor clinical decision making [2].

Several tools have been developed that assess the methodological quality of systematic reviews. One group of authors has attempted to create an easy to use quality assessment instrument, developed based on the most commonly used instruments in the published literature. This is known as AMSTAR and is a measurement tool to assess the methodological quality of systematic reviews [3]. This tool assesses review methodology across eleven relevant domains applying a ‘yes’, ‘no’, ‘n/a’ or ‘can't answer’ score to each domain. A study by the original authors has concluded that AMSTAR has good agreement, reliability, construct validity, and feasibility to assess the quality of systematic reviews, performing equally or better than similar tools in these areas. It also looks at methodological aspects not assessed by other instruments. In 2010, another group of researchers adapted AMSTAR to include a 4 point scale which was applied to each domain in order to produce a quantitative method of assessment, while retaining the validity of the original instrument [4]. The numerical data generated by R-AMSTAR allow the possibility of grading and ranking systematic reviews, which could assist clinicians who only want to select the ‘A’ graded reviews. It also permits an in-depth analysis of the quality of each domain, as well as of reviews as a whole.

We sought to assess the quality of a group of CRs and NCRs in the topic area of assisted reproduction. This topic was chosen because of the high number of reviews of assisted reproduction and as there is a need for high quality evidence for this expensive technology where live birth rates are generally no more than 30% for each started cycle. The objective of this project was to compare AMSTAR and R-AMSTAR on assessing systematic review quality of both Cochrane and non-Cochrane systematic reviews of assisted reproduction.

## Methods

### Inclusion criteria

All published systematic reviews on assisted reproductive technology with the latest search for studies taking place from 2007–2011 were considered for this project. We did not apply any language restrictions but each review had to include at least one study. We applied the Cochrane Collaboration definition of systematic reviews that is used by the Preferred Reporting Items for Systematic reviews and Meta-Analyses (PRISMA) statement: “The key characteristics of a systematic review are: (a) a clearly stated set of objectives with an explicit, reproducible methodology; (b) a systematic search that attempts to identify all studies that would meet the eligibility criteria; (c) an assessment of the validity of the findings of the included studies, for example through the assessment of risk of bias; and (d) systematic presentation, and synthesis, of the characteristics and findings of the included studies” [5]. Assisted reproduction included all steps in an assisted reproduction cycle but did not include intra-uterine insemination or ovulation induction in women with anovulation.

### Exclusion criteria

Reviews were not included in this study if they only had one RCT, if they considered diagnostic outcomes, or if they reported on interventions that were not considered to be assisted reproduction such as intra-uterine insemination (IUI) or ovulation induction for anovulatory women.

### Review selection

**Figure 1:**
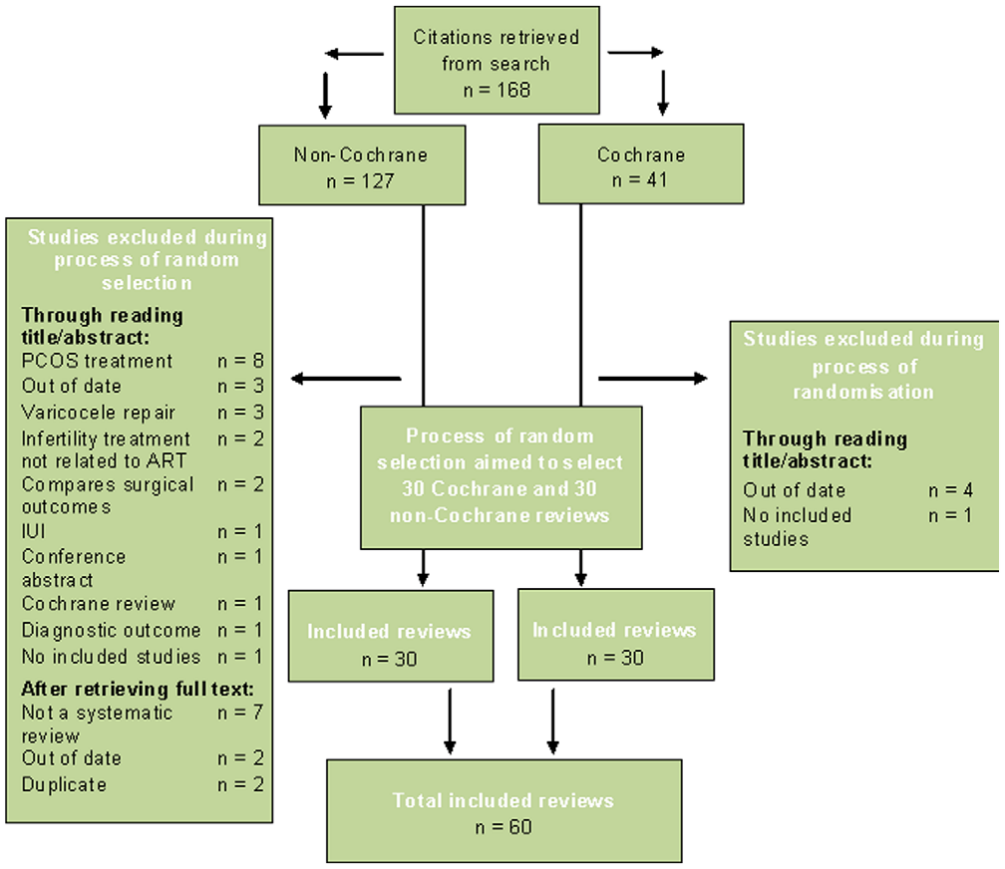
Process of study selection.

We searched the Cochrane Library for Cochrane reviews (CRs) on assisted reproduction for subfertility. The Cochrane Menstrual Disorders and Subfertility Group's PROCITE database of non Cochrane systematic reviews (NCRs) was searched in October 2011 for non-Cochrane systematic reviews on assisted reproduction. The search strategy can be found in Appendix S1. Two authors (IP, BW) randomly selected reviews from the resulting citations to form a list of thirty each of Cochrane and non-Cochrane reviews (see **Figure 1** for full details of the selection process). Any randomly selected reviews that did not meet the inclusion criteria, based on either full text or abstract, were excluded and a new review randomly selected to fill their place. Disagreements were resolved by discussion or consultation with a third author (CF).

### Search methods for identifying non Cochrane reviews

The scope of the MDSG register of systematic reviews includes review topics of menstrual disorders and subfertility. These reviews are sourced from weekly email alerts from MEDLINE (Ovid) and EMBASE (Ovid) databases. This register was searched from 01.01.07 to 27.10.11 using a selection of keyword and title field terms that included “assisted reproductive technologies” or “assisted reproductive technology” or “in vitro fertilization” (Appendix S1). There was no language restriction in this search.

The results from the search of the MDSG database were managed in an ENDNOTE library where duplications and inappropriate papers were removed.

### Assessment of methodological quality

The AMSTAR and the R-AMSTAR were applied to all 30 CRs and 30 NCRs (Appendices 3 and 4). The scores were converted to percentages, based on the maximum possible score (for the R-AMSTAR) and the number of domains with a ‘yes’ score (for the AMSTAR). Domains given a not-applicable (‘NA’) score were not used in the calculation. Based on the resulting percentage scores, grades were assigned to each review. This allowed comparison of the conclusions formed between the two assessment tools, using the surrogate measure of grades.

### Comparison of AMSTAR and r-AMSTAR

We calculated an overall agreement score using the weighted Cohen's Kappa, as well as one score for each item. For comparison of rating of methodological quality we calculated the chance-corrected agreement using kappa and chance independent agreement using PHI Φ. Kappa values of less than 0 rate as less than chance agreement; 0.01–0.20 slight agreement; 0.21–0.40 fair agreement; 0.41–0.60 moderate agreement; 0.61–0.80 substantial agreement; and 0.81–0.99 almost perfect agreement. We calculated PHI Φ for each question [6], [7].

A report comparing the differences between CRs and NCRs will be published separately. Our report focuses on the difference between AMSTAR and R-AMSTAR for reporting the quality of systematic reviews.

## Results

A description of the included reviews is presented in **Table 1**. These covered a range of interventions from all stages of an assisted reproductive technology cycle. There are notable differences between CRs and NCRs in their methodology including the number of data bases searched, and the reporting of the Boolean terms in the search, of excluded studies, of funding sources and of conflicts of interest.

**Table 1:** Epidemiology of Included Systematic Reviews.

Category	Characteristic	Cochrane (n = 30)		Non-Cochrane (n = 30)	
Year of publication *n (%)*	2006	2	(6.7)	0	(0.0)
	2007	3	(10.0)	1	(3.3)
	2008	4	(13.3)	5	(13.7)
	2009	4	(13.3)	9	(30.0)
	2010	8	(26.7)	10	(33.3)
	2011	9	(30.0)	5	(13.7)
Year of most recent search *n (%)*	2007	5	(13.7)	6	(20.0)
	2008	8	(26.7)	10	(33.3)
	2009	4	(13.3)	3	(10.0)
	2010	12	(40.0)	6	(20.0)
	2011	1	(3.3)	0	(0.0)
	Not given	0	(0.0)	5	(13.7)
Included Studies	Number, total	442		304	
	Median (IQR), per review	10	(4–18)	8	(6–13)
Number of databases searched	median (IQR)	8	(6–12.5)	4	(4–6)
Number of other sources searched median (IQR)	Median (IQR)	2.5	(2–4)	2	(1–2)
Years of coverage reported	N %	30	(100)	23	(77%)
Search terms reported as Full Boolean	N %	29	(97)	13	(43%)
Quality of primary studies assessed, component tool	N %	30	(100)	24	(80%)
Number of studies included, per review	median (IQR)	10	(4–18)		
Number of participants, per review	Median (IQR)	1504	(414–2508)		
Review flow reported	N (%)	30	(100)	29	(97)
Reasons for exclusion of studies reported	N (%)	30	(100)	28	(93)
Heterogeneity investigated (or intent to investigate)	N (%)	30	(100)	27	(90)
Pooling of studies	N (%)	27	(97)	28	(93)
Publication bias assessed (or intent to assess)	N (%)	17	(57)	11	(37)
Funding of review reported	N (%)	20	(67)	6	(20)
Funding of included studies reported	N (%)	16	(53)		
Conflict of interest reported	N (%)	30	(100)		

**Figure 2:**
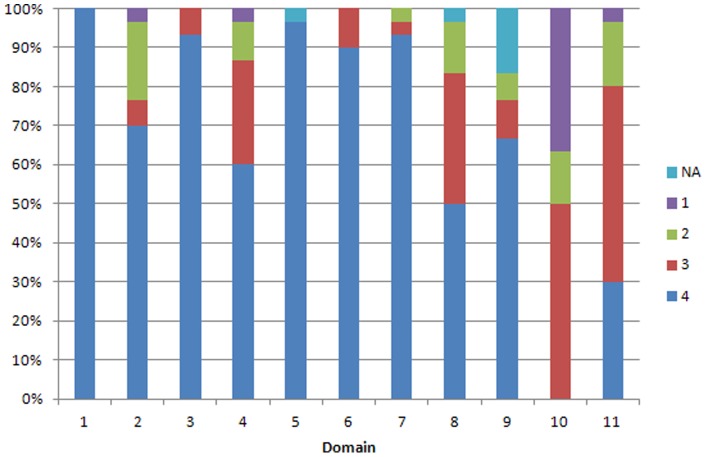
Performance of Cochrane reviews by R-AMSTAR domain. The graph shows the proportion of reviews for each possible R-AMSTAR score (1–4), for each domain, *where 1 is the lowest and 4 is the highest score*.

The findings of the AMSTAR and r-AMSTAR applied to the reviews are presented in **Table 2** and in **Figures 2** **,** **3** **,** **4** **,** **5**. Using the R-AMSTAR tool, 14 (46.7%) Cochrane reviews achieved an ‘A’ grade, 13 (43.3%) received a ‘B’ grade, and 3 (10%) a ‘C’ grade. According to AMSTAR, 12 (40%) Cochrane reviews were worthy of an ‘A’ grade, 12 (40%) of a ‘B’ grade, while 4 (13.3%) achieved ‘C’ grades, and 2 (6.7%) an ‘E’ grade.

**Figure 3:**
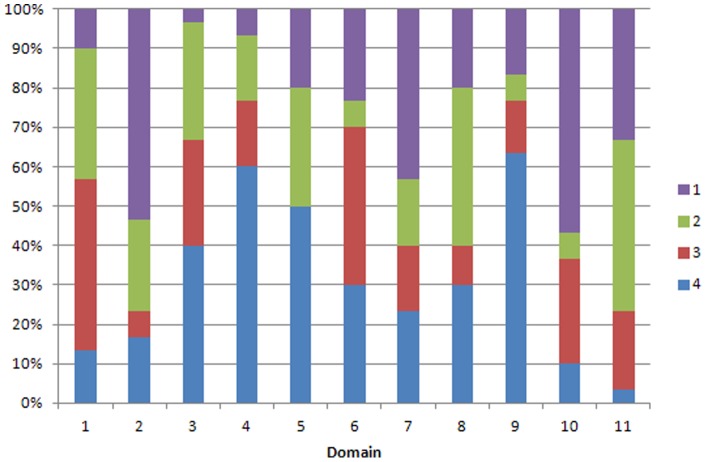
Performance of non-Cochrane reviews by R-AMSTAR domain. The graph shows the proportion of reviews for each possible R-AMSTAR score (1–4), for each domain, *where 1 is the lowest and 4 is the highest score*.

**Figure 4:**
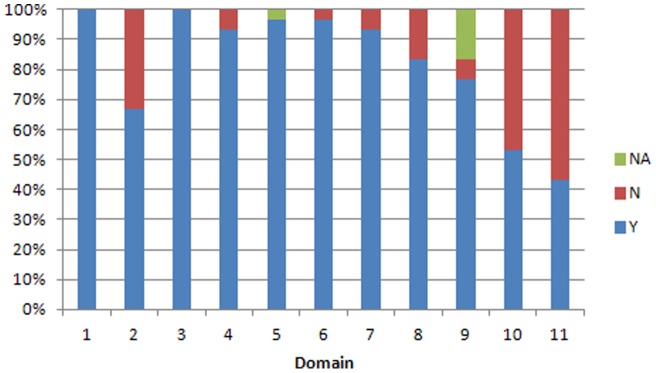
Performance of Cochrane reviews by domain in AMSTAR. *Y means the criteria are met; N means the criteria are not met; NA means not applicable*.

**Figure 5:**
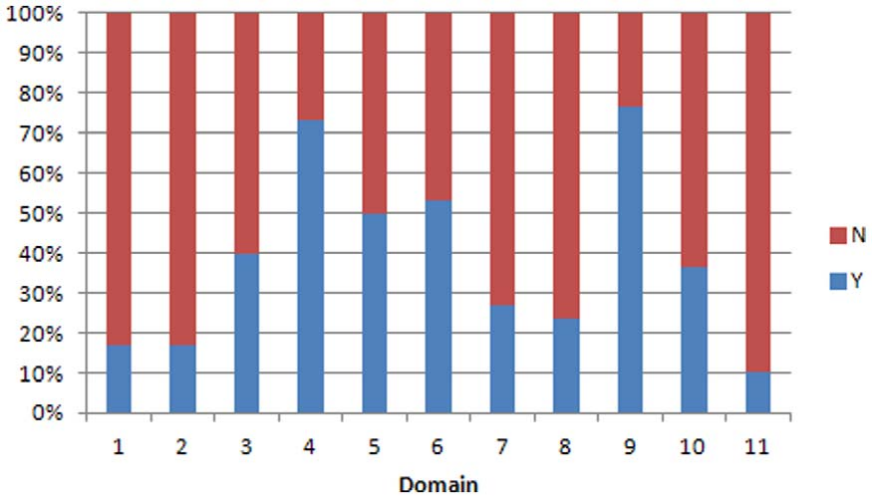
Performance of non-Cochrane reviews by domain in AMSTAR. *Y means the criteria are met; N means the criteria are not met*.

**Table 2:** Quality of the Cochrane and non-Cochrane Reviews according to AMSTAR and r-AMSTAR.

		Cochrane		Non-Cochrane	
		AMSTAR	r-AMSTAR	AMSTAR	r-AMSTAR
Grade*	A	12 (40%)	14 (46.7%)	0 (0%)	0 (0%)
	B	12 (40%)	13 (43.3%)	2 (6.7%)	3 (10%)
	C	4 (13.3%)	3 (10%)	1 (3.3%)	8 (26.7%)
	D	0 (0%)	0 (0%)	2 (6.7%)	8 (26.7%)
	E	2 (6.7%)	0 (0%)	5 (16.7%)	6 (20%)
	F	0 (0%)	0 (0%)	20 (66.7%)	5 (16.7%)

A; 90%, B; 80%, C; 70%, D; 60%, E; 50%, F; <50%.

By domain, CRs achieved perfect scores in domains 1 (*a priori* design) and 5 (list of included and excluded studies provided) by R-AMSTAR, and perfect scores in domains 1 (*a priori* design), 3 (comprehensive literature search), and 5 (list of included and excluded studies provided) by AMSTAR. According to both tools the worst performing domains were 10 (assessment of publication bias) and 11 (sources of support/conflicts of interest included).

For the NCRs, R-AMSTAR judged no review as deserving of an ‘A’ grade. 3 (10%) were given a ‘B’ grade, 8 (26.7%) a ‘C’ grade, 8 (26.7%) a ‘D’ grade, 6 (20%) an ‘E’ grade and 5 (16.7%) an ‘F’. In contrast, AMSTAR graded 2 (6.7%) reviews as ‘B’, 1 as ‘C’, 2 (6.7%) as ‘D’, 5 (16.7%) as ‘E’, and the rest (66.7%) ‘F’s.

By R-AMSTAR, NCRs performed best on domains 4 (inclusion of grey literature) and 9 (assessment of heterogeneity) and worst in domains 2 (independent data extraction and study selection), 10 (assessment of publication bias) and 11 (sources of support/conflicts of interest included). AMSTAR gave the best performance as also being in domains 4 and 9, but worst in domains 1 (a priori design), 2 and 11.

Applying the AMSTAR tool produced a much wider variation in percentage scores, as evidenced by the standard deviations. There was much less variation in the scores of CRs, with standard deviations of 5.5% and 11.5% (for R-AMSTAR and AMSTAR respectively), compared to 14.2% and 22.2% for NCRs. The total percentage score was fairly close between the two tools for CRs (88.3% vs. 83.6%; R-AMSTAR vs. AMSTAR), but quite disparate for NCRs, with a 63.9% score by R-AMSTAR and 38.5% by the AMSTAR.

**Figure 6:**
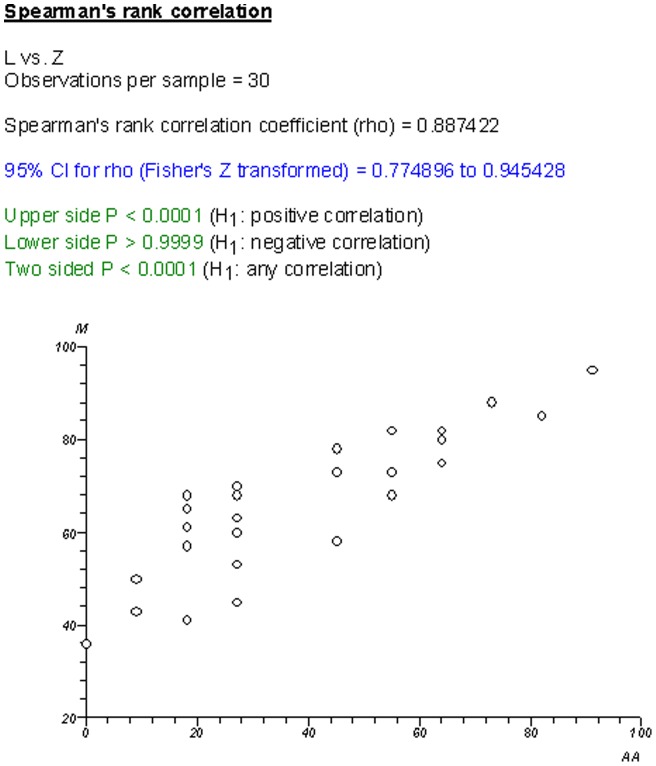
Non-Cochrane R-AMSTAR vs. non-Cochrane AMSTAR.

**Figure 7:**
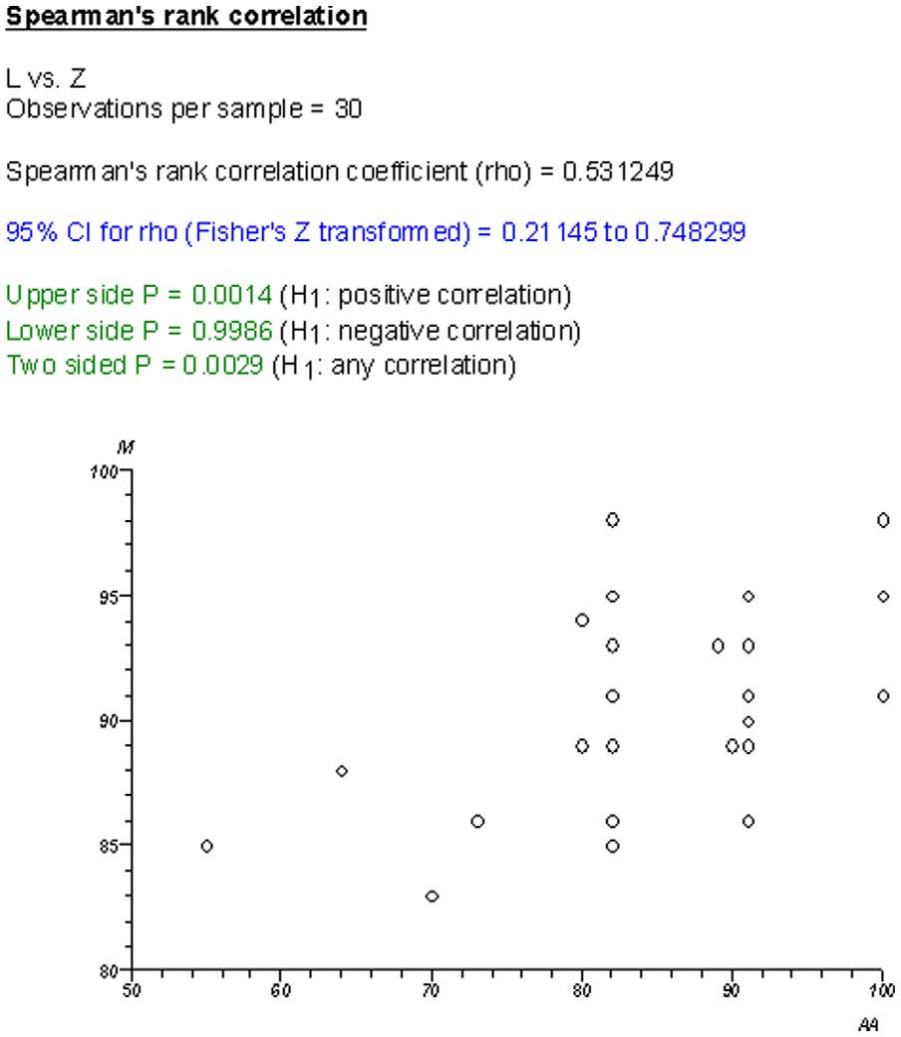
Cochrane R-AMSTAR vs. Cochrane AMSTAR.

There was poorer correlation between CRs than NCRs, as evidenced by the correlation graphs in **figures 6** and **7**. When comparing the ranking lists generated between AMSTAR and R-AMSTAR, CRs changed position by an average of 4.2 places while NCRs changed by an average of 2.9 places. The domains that were responsible for this in CRs were domains 8 (scientific quality of included studies used appropriately in formulating conclusions), 9 (assessment of heterogeneity), and 11 (sources of support/conflicts of interest included).

The 60 reviews included in this study had a wide range of quality scores. The overall scores estimated by the AMSTAR instrument ranged from 0 to 11 (out of a maximum score of 11) with a mean of 4.2 (95% CI: 3.3 to 5.1); median 4.0 (range 0 to 9.0) for NCRs and 9.0 (95% CL: 8.5 to 9.5); median 9.0 (6.0 to 11.0) for CRs. The overall scores for the R-AMSTAR ranged from 16 to 43 (out of a maximum score of 44) with a mean of 28.1 (95% CL: 25.9 to 30.3); median 29.5 (range 16.0 to 39.0) for NCRs and a mean of 38.0 (95% CL: 36.8 to 39.2); median 38.5 (range 29.0 to 43.0) for CRs.

Items in both AMSTAR and R-AMSTAR displayed levels of agreement that ranged from moderate to almost perfect. Using AMSTAR item 6 had a kappa of 0.52 (0.27 to 0.76), item 8 a kappa of 0.77 (0.52 to 1.02) and item 9 a kappa of 0.71 (0.53 to 0.88). All other items scored a kappa of >0.85. R-AMSTAR achieved lower kappas, with the lowest being item 4 with a kappa of 0.59 (0.42 to 0.77), item 6 with a kappa of 0.59 (0.40 to 0.78) and item 8 with a kappa of 0.45 (0.26 to 0.64). The full tables of scores can be found in **Tables 3** and **4**.

**Table 3:** Assessment of the inter-rater agreement for AMSTAR.

Items	Kappa (95% CI)	PHI Φ
1. Was an ‘a priori’ design provided?	Kappa = 1.0 (0.75 to 1.25)	Pi = 1.0
2. Was there duplicate study selection and data extraction?	Kappa = 0.93 (0.68 to 1.18)	Pi = 0.93
3. Was a comprehensive literature search performed?	Kappa = 1.0 (0.75 to 1.25)	Pi = 1.0
4. Was the status of publication (i.e. Grey literature) used as an inclusion criterion?	Kappa = 0.88 (0.63 to 1.13)	Pi = 0.88
5. Was a list of studies (included and excluded) provided?	Kappa = 1.0 (0.77 to 1.23)	Pi = 1.0
6. Were the characteristics of the included studies provided?	Kappa = 0. 52 (0.27 to 0.76)	Pi = 0.51
7. Was the scientific quality of the included studies assessed and documented?	Kappa = 0. 97(0.71 to 1.22)	Pi = 0.96
8. Was the scientific quality of the included studies used appropriately in formulating conclusion?	Kappa = 0.77 (0.52 to 1.02)	Pi = 0.77
9. Were the methods used to combine the findings of studies appropriate?	Kappa = 0.71 (0.53 to 0.88)	Pi = 0.69
10. Was the likelihood of publication bias assessed?	Kappa = 0.97 (0.71 to 1.22)	Pi = 0.97
11. Were potential conflicts of interest included?	Kappa = 0.87 (0.62 to 1.12)	Pi = 0.87

**Table 4:** Assessment of the inter-rater agreement for R-AMSTAR.

Items	Kappa (95% CI)	PHI Φ
Was an ‘a priori’ design provided?	Kappa = 0.70 (0.48 to 0.91)	Pi = 0.529412
Was there duplicate study selection and data extraction?	Kappa = 0.76 (0.55 to 0.98)	Pi = 0.67
Was a comprehensive literature search performed?	Kappa = 0.69 (0.51 to 0.88)	Pi = 0.58
Was the status of publication (ie. Grey literature) used as an inclusion criterion?	Kappa = 0.59 (0.42 to 0.77)	Pi = 0.60
Was a list of studies (included and excluded) provided?	Kappa = 0.81 (0.61 to 1.01)	Pi = 0.76
Were the characteristics of the included studies provided?	Kappa = 0.59 (0.40 to 0.78)	Pi = 0.52
Was the scientific quality of the included studies assessed and documented?	Kappa = 0.77 (0.57 to 0.97)	Pi = 0.58
Was the scientific quality of the included studies used appropriately in formulating conclusion?	Kappa = 0.45 (0.26 to 0.64)	Pi = 0.31
Were the methods used to combine the findings of studies appropriate?	Kappa = 0.67 (0.52 to 0.81)	Pi = 0.47
Was the likelihood of publication bias assessed?	Kappa = 0.87 (0.68 to 1.05)	Pi = 0.81
Were potential conflicts of interest included?	Kappa = 0.72 (0.55 to 0.89)	Pi = 0.61

## Discussion

The objective of this study was to compare two different approaches to assessing multiple systematic reviews. We have reported that R-AMSTAR and AMSTAR were similar but that the distribution of the scores was different. Both tools achieved near perfect consensus on the best and worst performing domains for each type of review. AMSTAR produced much greater variation in the total percentage scores, although the average score between the two tools was quite close for the set of Cochrane reviews, but dissimilar for non-Cochrane reviews. In spite of this similarity, there was poor correlation between the two tools for individual Cochrane reviews compared to non-Cochrane reviews.

The subjective nature of some domains presented challenges for the researchers. For example AMSTAR gave the criterion for a ‘yes’ in domain 8 as: ‘the results of the methodological rigor and scientific quality should be considered in the analysis and conclusions of the review, and explicitly stated in formulating recommendations’. R-AMSTAR split this criterion into two criteria (‘consideration of scientific quality’ and ‘explicit statement of quality in formulating recommendations’) and added two further criteria regarding a clinical consensus statement and whether this statement revises or confirms current practice guidelines. The sole AMSTAR criterion was difficult to apply as the judgement involved a great deal of subjectivity in deciding what constituted sufficient consideration of scientific quality. The R-AMSTAR criteria were perhaps even more difficult to apply as it was difficult to differentiate between criteria A and B, and between C and D (see Appendix S3). This impression is supported by kappa statistics which show poor inter rater reliability of domain 8 compared to the other domains. This was further complicated as AMSTAR domain 8 was also linked to domain 7 (assessment of scientific quality of included studies) in that a ‘no’ was automatically received for domain 8 if domain 7 was graded ‘no’. Scientific quality could still be adequately considered even it was not assessed by a quality tool and an *a priori* method. The combination of this with the subjectivity of this domain resulted in at least one occasion where a study received a ‘4’ by R-AMSTAR but a ‘no’ by AMSTAR for this domain, two obviously very divergent grades. A clinician looking at these two results for a review they are interested in would gain little insight into how well it performed in this area. Discrepancies in domain 8 were more common in the CRs and resulted in ranking being shifted significantly. The explanation for this is that CRs generally attempt to evaluate the effect of the quality of included studies on the conclusions drawn whereas NCRs more often did not. It was therefore easier to give consistently poor scores for the NCRs as any absence of an ‘attempt’ was easily noticed, whereas CRs might be judged favourably in their attempts once and unfavourably the next time.

Domain 11 was also difficult to apply. This domain considers the reporting of conflicts of interest. R-AMSTAR splits it into 3 criteria; statement of sources of support, absence of conflicts of interest, and assessment of sources of support/conflicts of interest in the studies included in the systematic review. A ‘yes’ for AMSTAR was equivalent to gaining the first and last of those criteria. Cochrane almost always included their sources of support and conflicts of interest but less commonly assessed conflicts of interest in their included studies, resulting in instances where a 3 would be given by R-AMSTAR but a ‘no’ by AMSTAR, which could result in shifts of ranking. In contrast NCRs were less reliable in assessing their conflicts of interest and sources of support, suggesting that the scores of the two tools should be more consistent with each other.

Discrepancies between the two approaches in non-Cochrane reviews were in domain 3 (comprehensive literature search), domain 6 (characteristics of included studies provided) and domain 7 (scientific quality of included studies assessed and documented). The problem with domain 3 was immediately obvious. To construct the R-AMSTAR criteria for this domain the components of the AMSTAR criterion were taken and split into 5 parts. Therefore a review would only be able to get a ‘yes’ by AMSTAR if the equivalent R-AMSTAR score was 4. This resulted in situations where R-AMSTAR gave a 3 but AMSTAR gave ‘no’, which could potentially be responsible for the ranking changes seen in NCRs. This would be more prevalent in NCRs as CRs performed very well in literature searching, very often receiving 4s.

It was domain 6, however, where the most discordancy appeared in these NCRs. The AMSTAR criterion required data from the original studies on participants, interventions, and outcomes and a range of characteristics reported e.g. age, race, sex, socioeconomic data, disease status, duration, severity, comorbidities, etc. R-AMSTAR separated these components into two criteria and added a third: whether the information on included studies on the whole appears to be complete and accurate. We conjecture that the discrepancies in this domain lie, as in domain 8, in the subjectivity of judgements, namely criterion B (range of relevant characteristics reported). It was difficult for us to judge what constituted a ‘range of relevant characteristics’. The third R-AMSTAR criterion also seemed to us to serve no particular purpose. The criterion itself is subjective, and in our assessment was invariably linked to performance in the other two criteria. Therefore, on initial assessment using R-AMSTAR, we could have judged a review not to have provided relevant characteristics, but reached the opposite view using AMSTAR. Assuming data on participants, interventions and outcomes were judged to be present both times, this would have resulted in a 2 from R-AMSTAR (neither criterion B or C given, see Appendix S3 for further information), but a ‘yes’ from AMSTAR. This is but one example of the way the subjectivity of this domain in both tools could lead to discordancy between them. That domain 6 was implicated equally in NCRs that both rose and fell in ranking lends evidence to the view that subjectivity is at fault.

Domain 7 mentioned the use of a quality scoring system, which the authors took to be a necessary criterion. Whether this was the intention of the note is unclear (see Appendix S4). Therefore to achieve a ‘yes’ for this domain required the provision of an *a priori* method to assess the scientific quality of the included studies and the use of a quality tool to evaluate this. R-AMSTAR contained 4 criteria for this domain, the two mentioned above, and in addition: the scientific quality of the included studies appearing to be meaningful and discussion/recognition/awareness of levels of evidence. In both instances of discrepancy in this domain, reviews were given a 3 by R-AMSTAR but a ‘no’ by AMSTAR. It is possible this is due to not meeting the ‘quality tool’ criterion, as NCRs that assessed quality of evidence often did not use a quality tool, but still met the other R-AMSTAR criteria (hence receiving a 3). Most CRs used a quality scoring tool.

Other concerns were noted with domain 2 (duplicate study selection and data extraction). This contained 3 criteria, the last two of which were: B) statement of recognition or awareness of consensus procedure for disagreements and C) disagreements among extractors resolved properly as stated or implied. The two criteria reflect each other as often a review would say, for example, ‘any disagreements were resolved by discussion or consultation with a third party’, taking care of both criteria at the same time. Thus, B and C were nearly always given either together or not at all. The only exception, occurring perhaps once or twice, was when a review stated there were no disagreements to resolve (giving criterion C but not B). We feel that criterion C is therefore redundant and unfairly inflates scores in this domain. Also in the R-AMSTAR, we encountered difficulty with domain 4 (status of publication used as inclusion criterion). The first two criteria overlapped. If the first criterion was met (‘the authors should state they searched for reports regardless of publication type‘), that implied that the second criterion was met also (‘the authors should state whether or not they excluded any reports based on their publication status, language etc’). If the authors did not state what kind of literature they searched for, neither of the first two criteria would be met. If the authors stated they excluded unpublished literature, they met the second criterion. This means a review that included all types of literature, but did not report this, would receive a lower mark than a review that explicitly stated they excluded grey literature. Essentially, a review was rewarded for pointing out poor methodology.

After considering this analysis we recommend that AMSTAR could be improved by providing greater clarity and guidance in its application and in what constitutes a met criterion, such as characteristics of included studies in domain 6. The quantification of R-AMSTAR is attractive, as well as its addition of the PICO research question in domain 1, language restrictions in domain 4, reasons for exclusion in domain 5, discussion of levels of evidence in domain 7 and conflicts of interest in domain 11. However, the duplication (e.g. domain 6) and subjectivity (e.g. domain 8) in R-AMSTAR limits its usefulness. The kappa statistics also suggest that it was more difficult to apply the r-AMSTAR tool consistently. In conclusion, we recommend that AMSTAR incorporates the findings of this study and produces additional guidance in order to improve its reliability and usefulness.

## Supporting Information

Appendix S1 **Search Strategy for Non-Cochrane Systematic Reviews.**
(DOCX)

Appendix S2 **Journals** (**and Impact Factor**) **of the Included Non-Cochrane Reviews.**
(DOCX)

Appendix S3 **R-AMSTAR quality assessment.**
(DOCX)

Appendix S4 **AMSTAR – a measurement tool to assess the methodological quality of systematic reviews.**
(DOCX)

Appendix S5 **Reference List of Included Cochrane Reviews.**
(DOCX)

Appendix S6 **Reference List of Included Non-Cochrane Reviews.**
(DOCX)

Appendix S7 **Individual R-AMSTAR scores for Included Cochrane Reviews.**
(DOCX)

Appendix S8 **Individual R-AMSTAR scores for Included Non-Cochrane Reviews.**
(DOCX)
