# Supplementary material for: Methodological Quality of Systematic Reviews in Subfertility: A Comparison of Two Different Approaches
Source: PLoS One. 2012 Dec 28;7(12):e50403. doi: 10.1371/journal.pone.0050403 (PMC3532502; doi:10.1371/journal.pone.0050403)
Supplement: Appendix S1 — Search Strategy for Non-Cochrane Systematic Reviews. (DOCX) [file pone.0050403.s001.docx]

**Appendix 1 Search Strategy for Non-Cochrane Systematic Reviews**

For non Cochrane reviews we searched the PROCITE Cochrane Menstrual Disorders and Subfertility Group (MDSG) database of non Cochrane reviews from 01.01.07 to 27.10.11 using the following keywords:

Keywords CONTAINS "IVF" or "in vitro fertilization" or "in-vitro fertilisation" or "ICSI" or "intracytoplasmic sperm injection" or "Embryo" or "in-vitro fertilization" or "preimplantation genetic screening" or "assisted reproductive technologies" or "assisted reproductive technology" or Title CONTAINS "IVF" or "in vitro fertilization" or "in-vitro fertilisation" or "ICSI" or "intracytoplasmic sperm injection" or "Embryo" or "in-vitro fertilization" or "preimplantation genetic screening" or "assisted reproductive technologies" or "assisted reproductive technology".
