# Supplementary material for: Methodological Quality of Systematic Reviews in Subfertility: A Comparison of Two Different Approaches
Source: PLoS One. 2012 Dec 28;7(12):e50403. doi: 10.1371/journal.pone.0050403 (PMC3532502; doi:10.1371/journal.pone.0050403)
Supplement: Appendix S2 — Journals (and Impact Factor) of the Included Non-Cochrane Reviews. (DOCX) [file pone.0050403.s002.docx]

**Appendix 2 Journals (and Impact Factor) of the**

**Included Non-Cochrane Reviews**

| **Journal** | **Impact Factor^1^** | **Included Reviews** |
| --- | --- | --- |
| Human Reproduction Update | 8.755 | AbdelHafez et al., 2009 Groeneveld et al., 2011 Kolibianakis et al., 2009 Martins et al., 2011 Youssef et al., 2010 |
| Fertility and Sterility | 3.958 | Bodri et al., 2011 Cobo & Diaz, 2011 Griesinger et al., 2008 Jee et al., 2010 |
| Human Reproduction | 4.357 | Kolibianakis et al., 2008 Papanikolaou et al., 2008 Sunkara et al., 2010 Wennerholm et al., 2009 |
| Reproductive BioMedicine Online | 2.285 | AbdelHafez et al., 2010 Al-Inany & Gelder, 2010 Al-Inany et al., 2008 Sunkara et al., 2011 |
| Reproductive Biology and Endocrinology | 1.695 | Baruffi et al., 2009 Lehert et al., 2010 Oliveira et al., 2010 |
| British Medical Journal | 13.471 | Manheimer et al., 2007 McLernon et al., 2010 |
| Biodrugs | 4.192 | Saz-Parkinson et al., 2009 |
| BJOG – An International Journal of Obstetrics and Gynecology | 3.349 | El-Toukhy et al., 2008 |
| Current Opinion in Obstetrics and Gynecology | 2.325 | El-Toukhy & Khalaf, 2009 |
| Expert Review of Obstetrics and Gynecology | unknown | Noble et al., 2010 |
| Gynecologic and Obstetric Investigation | 1.031 | Jee et al., 2009 |
| Gynecological Endocrinology | 1.461 | Al-Inany et al., 2009 |
| Human Fertility | unknown | Cheong et al., 2010 |
| Journal of Assisted Reproduction and Genetics | 1.253 | Checa et al., 2009 |
| ^1^Impact Factors can be found at http://admin-apps.webofknowledge.com/JCR/JCR? | | |
