# Supplementary material for: Methodological Quality of Systematic Reviews in Subfertility: A Comparison of Two Different Approaches
Source: PLoS One. 2012 Dec 28;7(12):e50403. doi: 10.1371/journal.pone.0050403 (PMC3532502; doi:10.1371/journal.pone.0050403)
Supplement: Appendix S3 — R-AMSTAR quality assessment. (DOCX) [file pone.0050403.s003.docx]

**Appendix 3 R-AMSTAR quality assessment**

1. **Was an ‘a priori’ design provided?**

If it satisfies 3 of the criteria →4

If it satisfies 2 of the criteria →3

If it satisfies 1 of the criteria →2

If it satisfies 0 of the criteria →1

Criteria:

| (A) ‘a priori’ design |
| --- |
| (B) statement of inclusion criteria |
| (C) PICO/PIPO research question (population, intervention, comparison, prediction, outcome) |

1. **Was there duplicate study selection and data extraction?**

If it satisfies 3 of the criteria →4

If it satisfies 3 of the criteria →3

If it satisfies 2 of the criteria →2

If it satisfies 0 of the criteria →1

Criteria:

| (A) There should be at least two independent data extractors as stated or implied. |
| --- |
| (B) Statement of recognition or awareness of consensus procedure for disagreements. |
| (C) Disagreements among extractors resolved properly as stated or implied |

1. **Was a comprehensive literature search performed?**

If it satisfies 4 or 5 of the criteria → 4

If it satisfies 3 of the criteria → 3

If it satisfies 2 of the criteria →2

If it satisfies 1 or 0 of the criteria → 1

Criteria:

| (A) At least two electronic sources should be searched. |
| --- |
| (B) The report must include years and databases used (e.g. Central, EMBASE, and MEDLINE). |
| (C) Key words and/or MESH terms must be stated **AND** where feasible the search strategy outline should be provided such that one can trace the filtering process of the included articles. |
| (D) In addition to the electronic databases (PubMed, EMBASE, Medline), all searches should be supplemented by consulting current contents, reviews, textbooks, specialized registers, or experts in the particular field of study, and by reviewing the references in the studies found. |
| (E) Journals were “hand-searched” or “manual searched” (i.e. identifying highly relevant journals and conducting a manual, page-by-page search of their entire contents looking for potentially eligible studies) |

1. **Was the status of publication (i.e. grey literature) used as an inclusion criterion?**

(Grey literature is literature produced at all levels of government, academia, business and industry in print and electronic formats, but is not controlled by commercial publishers. Examples can be but not limited to dissertations, conference proceedings.)

If it satisfies 3 of the criteria →4

If it satisfies 2 of the criteria →3

If it satisfies 1 of the criteria →2

If it satisfies 0 of the criteria →1

Criteria:

| (A) The authors should state that they searched for reports regardless of their publication type. |
| --- |
| (B) The authors should state whether or not they excluded any reports (from the systematic review), based on their publication status, language etc. |
| (C) “Non-English papers were translated” or readers sufficiently trained in foreign language |
| (D) No language restriction or recognition of non-English articles |

1. **Was a list of studies (included and excluded) provided?**

If it satisfies 4 of the criteria →4

If it satisfies 3 of the criteria →3

If it satisfies 2 of the criteria →2

If it satisfies 1 or 0 of the criteria → 1

Criteria:

| (A) Table/list/or figure of included studies, a reference list does not suffice. |
| --- |
| (B) Table/list/figure of excluded studies[^1^](http://www.ncbi.nlm.nih.gov/pmc/articles/PMC2948145/?tool=pubmed#FN1) either in the article or in a supplemental source (i.e. online). (Excluded studies refers to those studies seriously considered on the basis of title and/or abstract, but rejected after reading the body of the text) |
| (C) Author satisfactorily/sufficiently stated the reason for exclusion of the seriously considered studies. |
| (D) Reader is able to retrace the included and the excluded studies anywhere in the article bibliography, reference, or supplemental source |

1. **Were the characteristics of the included studies provided?**

If it satisfies 3 of the criteria →4

If it satisfies 2 of the criteria →3

If it satisfies 1 of the criteria →2

If it satisfies 0 criteria → 1

Criteria:

| (A) In an aggregated form such as a table, data from the original studies should be provided on the participants, interventions **AND** outcomes. |
| --- |
| (B) Provide the ranges of **relevant** characteristics in the studies analyzed (e.g. age, race, sex, relevant socioeconomic data, disease status, duration, severity, or other diseases should be reported.) |
| (C) The information provided appears to be complete and accurate (i.e. there is a tolerable range of subjectivity here. Is the reader left wondering? If so, state the needed information and the reasoning). |

1. **Was the scientific quality of the included studies assessed and documented?**

If it satisfies 4 of the criteria →4

If it satisfies 3 of the criteria →3

If it satisfies 2 of the criteria →2

If it satisfies 1 or 0 of the criteria →1

Criteria:

| (A) ‘A priori’ methods of assessment should be provided (e.g., for effectiveness studies if the author(s) chose to include only randomized, double-blind, placebo controlled studies, or allocation concealment as inclusion criteria); for other types of studies alternative items will be relevant. |
| --- |
| (B) The scientific quality of the included studies appears to be meaningful. |
| (C) Discussion/recognition/awareness of level of evidence |
| (D) Quality of evidence should be rated/ranked based on characterized instruments. (Characterized instrument is a created instrument that ranks the level of evidence, e.g. GRADE [Grading of Recommendations Assessment, Development and Evaluation.]) |

1. **Was the scientific quality of the included studies used appropriately in formulating conclusions?**

If it satisfies 4 of the criteria →4

If it satisfies 3 of the criteria →3

If it satisfies 2 of the criteria →2

If it satisfies 1 or 0 of the criteria → 1

Criteria:

| (A) The results of the methodological rigor and scientific quality should be considered in the analysis and the conclusions of the review |
| --- |
| (B) The results of the methodological rigor and scientific quality are **explicitly stated** in formulating recommendations. |
| (C) To have conclusions integrated/drives towards a clinical consensus statement |
| (D) This clinical consensus statement drives toward revision or confirmation of clinical practice guidelines |

1. **Were the methods used to combine the findings of studies appropriate?**

If it satisfy 4 of the criteria → 4

If it satisfy 3 of the criteria → 3

If it satisfy 2 of the criteria →2

If it satisfy 1 or 0 of the following criteria → 1

Criteria:

| (A) Statement of criteria that were used to decide that the studies analyzed were similar enough to be pooled? |
| --- |
| (B) For the pooled results, a test should be done to ensure the studies were combinable, to assess their homogeneity (i.e. Chi-squared test for homogeneity, I^2^). |
| (C) Is there a recognition of heterogeneity or lack of thereof |
| (D) If heterogeneity exists a “random effects model” should be used and/or the rationale (i.e. clinical appropriateness) of combining should be taken into consideration (i.e. is it sensible to combine?), or stated explicitly |
| (E) If homogeneity exists, author should state a rationale or a statistical test |

1. **Was the likelihood of publication bias (a.k.a. “file drawer” effect) assessed?**

If it satisfies 3 of the criteria →4

If it satisfies 2 of the criteria →3

If it satisfies 1 of the criteria →2

If it satisfies 0 of the criteria →1

Criteria:

| (A) Recognition of publication bias or file-drawer effect |
| --- |
| (B) An assessment of publication bias should include graphical aids (e.g., funnel plot, other available tests) |
| (C) Statistical tests (e.g., Egger regression test). |

1. **Was the conflict of interest stated?**

If it satisfies 3 of the criteria →4

If it satisfies 2 of the criteria →3

If it satisfies 1 of the criteria →2

If it satisfies 0 of the criteria →1

Criteria:

| (A) Statement of sources of support |
| --- |
| (B) No conflict of interest. This is subjective and may require some deduction or searching. |
| (C) An awareness/statement of support or conflict of interest in the **primary** inclusion studies |
