# Supplementary material for: Methodological Quality of Systematic Reviews in Subfertility: A Comparison of Two Different Approaches
Source: PLoS One. 2012 Dec 28;7(12):e50403. doi: 10.1371/journal.pone.0050403 (PMC3532502; doi:10.1371/journal.pone.0050403)
Supplement: Appendix S4 — AMSTAR – a measurement tool to assess the methodological quality of systematic reviews. (DOCX) [file pone.0050403.s004.docx]

**Appendix 4 AMSTAR - a measurement tool to assess
the methodological quality of systematic reviews**

| **AMSTAR – a measurement tool to assess the methodological quality of systematic reviews.** | |
| --- | --- |
| **1. Was an 'a priori' design provided?** The research question and inclusion criteria should be established before the conduct of the review.  *Note: Need to refer to a protocol, ethics approval, or pre-determined/a priori published research objectives to score a “yes.”* | □ Yes □ No □ Can't answer □ Not applicable |
|  | |
| **2. Was there duplicate study selection and data extraction?** There should be at least two independent data extractors and a consensus procedure for disagreements should be in place.  *Note: 2 people do study selection, 2 people do data extraction, consensus process or one person checks the other’s work.* | □ Yes □ No □ Can't answer □ Not applicable |
|  | |
| **3. Was a comprehensive literature search performed?** At least two electronic sources should be searched. The report must include years and databases used (e.g., Central, EMBASE, and MEDLINE). Key words and/or MESH terms must be stated and where feasible the search strategy should be provided. All searches should be supplemented by consulting current contents, reviews, textbooks, specialized registers, or experts in the particular field of study, and by reviewing the references in the studies found.  *Note: If at least 2 sources + one supplementary strategy used, select “yes” (Cochrane register/Central counts as 2 sources; a grey literature search counts as supplementary).* | □ Yes □ No □ Can't answer □ Not applicable |
|  | |
| **4. Was the status of publication (i.e. grey literature) used as an inclusion criterion?** The authors should state that they searched for reports regardless of their publication type. The authors should state whether or not they excluded any reports (from the systematic review), based on their publication status, language etc.  *Note: If review indicates that there was a search for “grey literature” or “unpublished literature,” indicate “yes.” SIGLE database, dissertations, conference proceedings, and trial registries are all considered grey for this purpose. If searching a source that contains both grey and non-grey, must specify that they were searching for grey/unpublished lit.* | □ Yes □ No □ Can't answer □ Not applicable |
|  | |
| **5. Was a list of studies (included and excluded) provided?** A list of included and excluded studies should be provided.  *Note: Acceptable if the excluded studies are referenced. If there is an electronic link to the list but the link is dead, select “no.”* | □ Yes □ No □ Can't answer □ Not applicable |
|  | |
| **6. Were the characteristics of the included studies provided?** In an aggregated form such as a table, data from the original studies should be provided on the participants, interventions and outcomes. The ranges of characteristics in all the studies analyzed e.g., age, race, sex, relevant socioeconomic data, disease status, duration, severity, or other diseases should be reported.  *Note: Acceptable if not in table format as long as they are described as above.* | □ Yes □ No □ Can't answer □ Not applicable |
|  | |
| **7. Was the scientific quality of the included studies assessed and documented?** 'A priori' methods of assessment should be provided (e.g., for effectiveness studies if the author(s) chose to include only randomized, double-blind, placebo controlled studies, or allocation concealment as inclusion criteria); for other types of studies alternative items will be relevant.  *Note: Can include use of a quality scoring tool or checklist, e.g., Jadad scale, risk of bias, sensitivity analysis, etc., or a description of quality items, with some kind of result for EACH study (“low” or “high” is fine, as long as it is clear which studies scored “low” and which scored “high”; a summary score/range for all studies is not acceptable).* | □ Yes □ No □ Can't answer □ Not applicable |
|  | |
| **8. Was the scientific quality of the included studies used appropriately in formulating conclusions?** The results of the methodological rigor and scientific quality should be considered in the analysis and the conclusions of the review, and explicitly stated in formulating recommendations.  *Note: Might say something such as “the results should be interpreted with caution due to poor quality of included studies.” Cannot score “yes” for this question if scored “no” for question 7.* | □ Yes □ No □ Can't answer □ Not applicable |
|  | |
| **9. Were the methods used to combine the findings of studies appropriate?** For the pooled results, a test should be done to ensure the studies were combinable, to assess their homogeneity (i.e., Chi-squared test for homogeneity, I^2^). If heterogeneity exists a random effects model should be used and/or the clinical appropriateness of combining should be taken into consideration (i.e., is it sensible to combine?).  *Note: Indicate “yes” if they mention or describe heterogeneity, i.e., if they explain that they cannot pool because of heterogeneity/variability between interventions.* | □ Yes □ No □ Can't answer □ Not applicable |
|  | |
| **10. Was the likelihood of publication bias assessed?** An assessment of publication bias should include a combination of graphical aids (e.g., funnel plot, other available tests) and/or statistical tests (e.g., Egger regression test, Hedges-Olken).  *Note: If no test values or funnel plot included, score “no”. Score “yes” if mentions that publication bias could not be assessed because there were fewer than 10 included studies.* | □ Yes □ No □ Can't answer □ Not applicable |
|  | |
| **11. Was the conflict of interest included?** Potential sources of support should be clearly acknowledged in both the systematic review and the included studies.  *Note: To get a “yes,” must indicate source of funding or support for the systematic review AND for each of the included studies.* | □ Yes □ No □ Can't answer □ Not applicable |
|  | |
| Shea et al. BMC Medical Research Methodology 2007 **7**:10   doi:10.1186/1471-2288-7-10 | |
