# Supplementary material for: Methodological Quality of Systematic Reviews in Subfertility: A Comparison of Two Different Approaches
Source: PLoS One. 2012 Dec 28;7(12):e50403. doi: 10.1371/journal.pone.0050403 (PMC3532502; doi:10.1371/journal.pone.0050403)
Supplement: Appendix S5 — Reference List of Included Cochrane Reviews. (DOCX) [file pone.0050403.s005.docx]

**Appendix 5 Reference List of Included Cochrane Reviews**

1. Abou-Setta AM, D'Angelo A, Sallam HN, Hart RJ, Al-Inany HG. Post-embryo transfer interventions for in vitro fertilization and intracytoplasmic sperm injection patients. Cochrane Database of Systematic Reviews [serial on the Internet]. 2009; (4): Available from: http://www.mrw.interscience.wiley.com/cochrane/clsysrev/articles/CD006567/frame.html.

2. Al-Inany HG, Youssef MAFM, Aboulghar M, Broekmans FJ, Sterrenburg MD, Smit JG, et al. Gonadotrophin-releasing hormone antagonists for assisted reproductive technology. Cochrane Database of Systematic Reviews [serial on the Internet]. 2011; (5): Available from: http://www.mrw.interscience.wiley.com/cochrane/clsysrev/articles/CD001750/frame.html.

3. Anderson K, Norman RJ, Middleton P. Preconception lifestyle advice for people with subfertility. Cochrane Database of Systematic Reviews [serial on the Internet]. 2010; (4): Available from: http://www.mrw.interscience.wiley.com/cochrane/clsysrev/articles/CD008189/frame.html.

4. Benschop L, Farquhar C, van der Poel N, Heineman MJ. Interventions for women with endometrioma prior to assisted reproductive technology. Cochrane Database of Systematic Reviews [serial on the Internet]. 2010; (11).

5. Blake D, Farquhar C, Johnson N, Proctor M. Cleavage stage versus blastocyst stage embryo transfer in assisted reproductive technology. Cochrane Database of Systematic Reviews [serial on the Internet]. 2007; (4): Available from: http://www.mrw.interscience.wiley.com/cochrane/clsysrev/articles/CD002118/frame.html.

6. Boomsma CM, Keay SD, Macklon NS. Peri-implantation glucocorticoid administration for assisted reproductive technology cycles. Cochrane Database of Systematic Reviews [serial on the Internet]. 2007; (1): Available from: http://www.mrw.interscience.wiley.com/cochrane/clsysrev/articles/CD005996/frame.html.

7. Brown J, Buckingham K, Abou-Setta AM, Buckett W. Ultrasound versus 'clinical touch' for catheter guidance during embryo transfer in women. Cochrane Database of Systematic Reviews [serial on the Internet]. 2010; (1): Available from: http://www.mrw.interscience.wiley.com/cochrane/clsysrev/articles/CD006107/frame.html.

8. Cheong YC, Hung Yu Ng E, Ledger WL. Acupuncture and assisted conception. Cochrane Database of Systematic Reviews [serial on the Internet]. 2008; (4): Available from: http://www.mrw.interscience.wiley.com/cochrane/clsysrev/articles/CD006920/frame.html.

9. D'Angelo A, Amso NN. Embryo freezing for preventing ovarian hyperstimulation syndrome. Cochrane Database of Systematic Reviews [serial on the Internet]. 2007; (3): Available from: http://www.mrw.interscience.wiley.com/cochrane/clsysrev/articles/CD002806/frame.html.

10. D'Angelo A, Brown J, Amso NN. Coasting (withholding gonadotrophins) for preventing ovarian hyperstimulation syndrome. Cochrane Database of Systematic Reviews [serial on the Internet]. 2011; (6): Available from: http://www.mrw.interscience.wiley.com/cochrane/clsysrev/articles/CD002811/frame.html.

11. Derks RS, Farquhar C, Mol BWJ, Buckingham K, Heineman MJ. Techniques for preparation prior to embryo transfer. Cochrane Database of Systematic Reviews [serial on the Internet]. 2009; (4): Available from: http://www.mrw.interscience.wiley.com/cochrane/clsysrev/articles/CD007682/frame.html.

12. Duffy JMN, Ahmad G, Mohiyiddeen L, Nardo LG, Watson A. Growth hormone for in vitro fertilization. Cochrane Database of Systematic Reviews [serial on the Internet]. 2010; (1): Available from: http://www.mrw.interscience.wiley.com/cochrane/clsysrev/articles/CD000099/frame.html.

13. Ghobara T, Vanderkerchove P. Cycle regimens for frozen-thawed embryo transfer. Cochrane Database of Systematic Reviews [serial on the Internet]. 2008; (1): Available from: http://www.mrw.interscience.wiley.com/cochrane/clsysrev/articles/CD003414/frame.html.

14. Glujovsky D, Pesce R, Fiszbajn G, Sueldo C, Hart RJ, Ciapponi A. Endometrial preparation for women undergoing embryo transfer with frozen embryos or embryos derived from donor oocytes. Cochrane Database of Systematic Reviews [serial on the Internet]. 2010; (1): Available from: http://www.mrw.interscience.wiley.com/cochrane/clsysrev/articles/CD006359/frame.html.

15. Kwan I, Bhattacharya S, McNeil A. Monitoring of stimulated cycles in assisted reproduction (IVF and ICSI). Cochrane Database of Systematic Reviews [serial on the Internet]. 2008; (2): Available from: http://www.mrw.interscience.wiley.com/cochrane/clsysrev/articles/CD005289/frame.html.

16. Maheshwari A, Gibreel A, Siristatidis CS, Bhattacharya S. Gonadotrophin-releasing hormone agonist protocols for pituitary suppression in assisted reproduction. Cochrane Database of Systematic Reviews [serial on the Internet]. 2011; (8): Available from: http://www.mrw.interscience.wiley.com/cochrane/clsysrev/articles/CD006919/frame.html.

17. Pandian Z, Bhattacharya S, Ozturk O, Serour GI, Templeton A. Number of embryos for transfer following in-vitro fertilisation or intra-cytoplasmic sperm injection. Cochrane Database of Systematic Reviews [serial on the Internet]. 2009; (2): Available from: http://www.mrw.interscience.wiley.com/cochrane/clsysrev/articles/CD003416/frame.html.

18. Pandian Z, McTavish AR, Aucott L, Hamilton MPR, Bhattacharya S. Interventions for 'poor responders' to controlled ovarian hyper stimulation (COH) in in-vitro fertilisation (IVF). Cochrane Database of Systematic Reviews [serial on the Internet]. 2010; (1): Available from: http://www.mrw.interscience.wiley.com/cochrane/clsysrev/articles/CD004379/frame.html.

19. Proctor M, Johnson N, van Peperstraten AM, Phillipson G. Techniques for surgical retrieval of sperm prior to intra-cytoplasmic sperm injection (ICSI) for azoospermia. Cochrane Database of Systematic Reviews [serial on the Internet]. 2008; (2).

20. Showell MG, Brown J, Yazdani A, Stankiewicz MT, Hart RJ. Antioxidants for male subfertility. Cochrane Database of Systematic Reviews [serial on the Internet]. 2011; (1): Available from: http://www.mrw.interscience.wiley.com/cochrane/clsysrev/articles/CD007411/frame.html.

21. Smulders B, van Oirschot SM, Farquhar C, Rombauts L, Kremer JAM. Oral contraceptive pill, progestogen or estrogen pre-treatment for ovarian stimulation protocols for women undergoing assisted reproductive techniques. Cochrane Database of Systematic Reviews [serial on the Internet]. 2010; (1): Available from: http://www.mrw.interscience.wiley.com/cochrane/clsysrev/articles/CD006109/frame.html.

22. Tso LO, Costello MF, Andriolo RB, Freitas V. Metformin treatment before and during IVF or ICSI in women with polycystic ovary syndrome. Cochrane Database of Systematic Reviews [serial on the Internet]. 2009; (2): Available from: http://www.mrw.interscience.wiley.com/cochrane/clsysrev/articles/CD006105/frame.html.

23. Twisk M, Mastenbroek S, van Wely M, Heineman MJ, Van der Veen F, Repping S. Preimplantation genetic screening for abnormal number of chromosomes (aneuploidies) in in vitro fertilisation or intracytoplasmic sperm injection. Cochrane Database of Systematic Reviews [serial on the Internet]. 2006; (1): Available from: http://www.mrw.interscience.wiley.com/cochrane/clsysrev/articles/CD005291/frame.html.

24. van der Linden M, Buckingham K, Farquhar C, Kremer JAM, Metwally M. Luteal phase support for assisted reproduction cycles. Cochrane Database of Systematic Reviews [serial on the Internet]. 2011; (10): Available from: http://www.mrw.interscience.wiley.com/cochrane/clsysrev/articles/CD009154/frame.html.

25. van Rumste MME, Evers JLH, Farquhar C. Intra-cytoplasmic sperm injection versus conventional techniques for oocyte insemination during in vitro fertilisation in couples with non-male subfertility. Cochrane Database of Systematic Reviews [serial on the Internet]. 2003; (2): Available from: http://www.mrw.interscience.wiley.com/cochrane/clsysrev/articles/CD001301/frame.html.

26. van Wely M, Kwan I, Burt AL, Thomas J, Vail A, Van der Veen F, et al. Recombinant versus urinary gonadotrophin for ovarian stimulation in assisted reproductive technology cycles. Cochrane Database of Systematic Reviews [serial on the Internet]. 2011; (2): Available from: http://www.mrw.interscience.wiley.com/cochrane/clsysrev/articles/CD005354/frame.html.

27. Wongtra-ngan S, Vutyavanich T, Brown J. Follicular flushing during oocyte retrieval in assisted reproductive techniques. Cochrane Database of Systematic Reviews [serial on the Internet]. 2010; (9): Available from: http://www.mrw.interscience.wiley.com/cochrane/clsysrev/articles/CD004634/frame.html.

28. Youssef MAFM, Al-Inany HG, Aboulghar M, Mansour R, Abou-Setta AM. Recombinant versus urinary human chorionic gonadotrophin for final oocyte maturation triggering in IVF and ICSI cycles. Cochrane Database of Systematic Reviews [serial on the Internet]. 2011; (4): Available from: http://www.mrw.interscience.wiley.com/cochrane/clsysrev/articles/CD003719/frame.html.

29. Youssef MAFM, Al-Inany HG, Evers JLH, Aboulghar M. Intra-venous fluids for the prevention of severe ovarian hyperstimulation syndrome. Cochrane Database of Systematic Reviews [serial on the Internet]. 2011; (2): Available from: http://www.mrw.interscience.wiley.com/cochrane/clsysrev/articles/CD001302/frame.html.

30. Youssef MAFM, Van der Veen F, Al-Inany HG, Griesinger G, Mochtar MH, Aboulfoutouh I, et al. Gonadotropin-releasing hormone agonist versus HCG for oocyte triggering in antagonist assisted reproductive technology cycles. Cochrane Database of Systematic Reviews [serial on the Internet]. 2011; (1): Available from: http://www.mrw.interscience.wiley.com/cochrane/clsysrev/articles/CD008046/frame.html.
