# Supplementary material for: Methodological Quality of Systematic Reviews in Subfertility: A Comparison of Two Different Approaches
Source: PLoS One. 2012 Dec 28;7(12):e50403. doi: 10.1371/journal.pone.0050403 (PMC3532502; doi:10.1371/journal.pone.0050403)
Supplement: Appendix S6 — Reference List of Included Non-Cochrane Reviews. (DOCX) [file pone.0050403.s006.docx]

**Appendix 6 Reference List of Included Non-Cochrane Reviews**

1. AbdelHafez F, Bedaiwy M, El-Nashar SA, Sabanegh E, Desai N. Techniques for cryopreservation of individual or small numbers of human spermatozoa: a systematic review. Hum Reprod Update. [Review]. 2009 Mar-Apr;15(2):153-64.

2. AbdelHafez FF, Desai N, Abou-Setta AM, Falcone T, Goldfarb J. Slow freezing, vitrification and ultra-rapid freezing of human embryos: a systematic review and meta-analysis. Reproductive BioMedicine Online. [Comparative Study Meta-Analysis

Review]. 2010 Feb;20(2):209-22.

3. Al-Inany HG, Abou-Setta AM, Aboulghar MA, Mansour RT, Serour GI. Efficacy and safety of human menopausal gonadotrophins versus recombinant FSH: a meta-analysis. Reproductive Biomedicine Online. 2008 Jan;16(1):81-8.

4. Al-Inany HG, Abou-Setta AM, Aboulghar MA, Mansour RT, Serour GI. Highly purified hMG achieves better pregnancy rates in IVF cycles but not ICSI cycles compared with recombinant FSH: a meta-analysis. Gynecological endocrinology. [Comparative Study Meta-Analysis]. 2009 Jun;25(6):372-8.

5. Al-Inany HG, van Gelder P. Effect of urinary versus recombinant FSH on clinical outcomes after frozen-thawed embryo transfers: a systematic review. Reproductive BioMedicine Online. [Research Support, Non-U.S. Gov't Review]. 2010 Aug;21(2):151-8.

6. Baruffi RL, Mauri AL, Petersen CG, Nicoletti A, Pontes A, Oliveira JB, et al. Single-embryo transfer reduces clinical pregnancy rates and live births in fresh IVF and Intracytoplasmic Sperm Injection (ICSI) cycles: a meta-analysis. Reproductive Biology & Endocrinology. 2009;7:36.

7. Bodri D, Sunkara SK, Coomarasamy A. Gonadotropin-releasing hormone agonists versus antagonists for controlled ovarian hyperstimulation in oocyte donors: a systematic review and meta-analysis. Fertil Steril. [Comparative Study Meta-Analysis

Review]. 2011 Jan;95(1):164-9.

8. Checa MA, Alonso-Coello P, Sola I, Robles A, Carreras R, Balasch J. IVF/ICSI with or without preimplantation genetic screening for aneuploidy in couples without genetic disorders: a systematic review and meta-analysis. J Assist Reprod Genet. [Meta-Analysis Review]. 2009 May;26(5):273-83.

9. Cheong Y, Nardo LG, Rutherford T, Ledger W. Acupuncture and herbal medicine in in vitro fertilisation: a review of the evidence for clinical practice. Hum Fertil (Camb). [Meta-Analysis]. 2010 Mar;13(1):3-12.

10. Cobo A, Diaz C. Clinical application of oocyte vitrification: a systematic review and meta-analysis of randomized controlled trials. Fertil Steril. [Meta-Analysis Review]. 2011 Aug;96(2):277-85.

11. El-Toukhy T, Khalaf Y. The impact of acupuncture on assisted reproductive technology outcome. Curr Opin Obstet Gynecol. [Meta-Analysis Review]. 2009 Jun;21(3):240-6.

12. El-Toukhy T, Sunkara SK, Khairy M, Dyer R, Khalaf Y, Coomarasamy A. A systematic review and meta-analysis of acupuncture in in vitro fertilisation. Bjog. [Meta-Analysis Review]. 2008 Sep;115(10):1203-13.

13. Griesinger G, Venetis CA, Marx T, Diedrich K, Tarlatzis BC, Kolibianakis EM. Oral contraceptive pill pretreatment in ovarian stimulation with GnRH antagonists for IVF: a systematic review and meta-analysis. Fertil Steril. [Meta-Analysis Review]. 2008 Oct;90(4):1055-63.

14. Groeneveld E, Broeze KA, Lambers MJ, Haapsamo M, Dirckx K, Schoot BC, et al. Is aspirin effective in women undergoing in vitro fertilization (IVF)? Results from an individual patient data meta-analysis (IPD MA). Hum Reprod Update. 2011 Jul-Aug;17(4):501-9.

15. Jee BC, Suh CS, Kim SH. Ectopic pregnancy rates after frozen versus fresh embryo transfer: a meta-analysis. Gynecol Obstet Invest. [Meta-Analysis]. 2009;68(1):53-7.

16. Jee BC, Suh CS, Kim SH, Kim YB, Moon SY. Effects of estradiol supplementation during the luteal phase of in vitro fertilization cycles: a meta-analysis. Fertility & Sterility. 2010 Feb;93(2):428-36.

17. Kolibianakis EM, Venetis CA, Diedrich K, Tarlatzis BC, Griesinger G. Addition of growth hormone to gonadotrophins in ovarian stimulation of poor responders treated by in-vitro fertilization: a systematic review and meta-analysis. Hum Reprod Update. [Meta-Analysis Review]. 2009 Nov-Dec;15(6):613-22.

18. Kolibianakis EM, Venetis CA, Papanikolaou EG, Diedrich K, Tarlatzis BC, Griesinger G. Estrogen addition to progesterone for luteal phase support in cycles stimulated with GnRH analogues and gonadotrophins for IVF: a systematic review and meta-analysis. Human Reproduction. [Meta-Analysis Review]. 2008 Jun;23(6):1346-54.

19. Lehert P, Schertz JC, Ezcurra D. Recombinant human follicle-stimulating hormone produces more oocytes with a lower total dose per cycle in assisted reproductive technologies compared with highly purified human menopausal gonadotrophin: a meta-analysis. Reprod Biol Endocrinol. [Comparative Study Meta-Analysis

Review]. 2010;8:112.

20. Manheimer E, Zhang G, Udoff L, Haramati A, Langenberg P, Berman BM, et al. Effects of acupuncture on rates of pregnancy and live birth among women undergoing in vitro fertilisation: systematic review and meta-analysis. Bmj. [Meta-Analysis

Research Support, N.I.H., Extramural Review]. 2008 Mar 8;336(7643):545-9.

21. Martins WP, Rocha IA, Ferriani RA, Nastri CO. Assisted hatching of human embryos: a systematic review and meta-analysis of randomized controlled trials. Hum Reprod Update. [Meta-Analysis Review]. 2011 Jul-Aug;17(4):438-53.

22. McLernon DJ, Harrild K, Bergh C, Davies MJ, de Neubourg D, Dumoulin JCM, et al. Clinical effectiveness of elective single versus double embryo transfer: meta-analysis of individual patient data from randomised trials. Bmj. 2010;341(dec21 2):c6945-c.

23. Noble B, Harvey C, Napier C, Curtis-Barton M, McGarraghy M, Maheshwari A. Preimplantation genetic screening in advanced maternal age: a systematic review. Expert Reviews of Obstetrics and Gynecology [serial on the Internet]. 2010; 5(6).

24. Oliveira JB, Baruffi R, Petersen CG, Mauri AL, Cavagna M, Franco JG. Administration of single-dose GnRH agonist in the luteal phase in ICSI cycles: a meta-analysis. Reprod Biol Endocrinol. [Meta-Analysis Review]. 2010;8:107.

25. Papanikolaou EG, Kolibianakis EM, Tournaye H, Venetis CA, Fatemi H, Tarlatzis B, et al. Live birth rates after transfer of equal number of blastocysts or cleavage-stage embryos in IVF. A systematic review and meta-analysis. Human Reproduction. [Meta-Analysis Review]. 2008 Jan;23(1):91-9.

26. Saz-Parkinson Z, Lopez-Cuadrado T, Bouza C, Amate JM. Outcomes of new quality standards of follitropin alfa on ovarian stimulation. Meta-analysis of previous studies. BioDrugs. 2009;23(1):37-42.

27. Sunkara SK, Pundir J, Khalaf Y. Effect of androgen supplementation or modulation on ovarian stimulation outcome in poor responders: a meta-analysis. Reproductive BioMedicine Online. 2011 Jun;22(6):545-55.

28. Sunkara SK, Siozos A, Bolton VN, Khalaf Y, Braude PR, El-Toukhy T. The influence of delayed blastocyst formation on the outcome of frozen-thawed blastocyst transfer: a systematic review and meta-analysis. Human Reproduction. [Meta-Analysis Review]. 2010 Aug;25(8):1906-15.

29. Wennerholm UB, Soderstrom-Anttila V, Bergh C, Aittomaki K, Hazekamp J, Nygren KG, et al. Children born after cryopreservation of embryos or oocytes: a systematic review of outcome data. Human Reproduction. [Research Support, Non-U.S. Gov't

Review]. 2009 Sep;24(9):2158-72.

30. Youssef MAFM, van Wely M, Hassan MA, Al-Inany HG, Mochtar M, Khattab S, et al. Can dopamine agonists reduce the incidence and severity of OHSS in IVF/ICSI treatment cycles? A systematic review and meta-analysis. Hum Reprod Update. [Meta-Analysis Review]. 2010 Sep-Oct;16(5):459-66.
