# Supplementary material for: Methodological Quality of Systematic Reviews in Subfertility: A Comparison of Two Different Approaches
Source: PLoS One. 2012 Dec 28;7(12):e50403. doi: 10.1371/journal.pone.0050403 (PMC3532502; doi:10.1371/journal.pone.0050403)
Supplement: Appendix S7 — Individual R-AMSTAR scores for Included Cochrane Reviews. (DOCX) [file pone.0050403.s007.docx]

**Appendix 7 Individual R-AMSTAR scores for Included Cochrane Reviews**
