# Supplementary material for: Methodological Quality of Systematic Reviews in Subfertility: A Comparison of Two Different Approaches
Source: PLoS One. 2012 Dec 28;7(12):e50403. doi: 10.1371/journal.pone.0050403 (PMC3532502; doi:10.1371/journal.pone.0050403)
Supplement: Appendix S8 — Individual R-AMSTAR scores for Included Non-Cochrane Reviews. (DOCX) [file pone.0050403.s008.docx]

**Appendix 8 Individual R-AMSTAR scores for**

**Included Non-Cochrane Reviews**
